# Hyaluronic acid-modified manganese-chelated dendrimer-entrapped gold nanoparticles for the targeted CT/MR dual-mode imaging of hepatocellular carcinoma

**DOI:** 10.1038/srep33844

**Published:** 2016-09-22

**Authors:** Ruizhi Wang, Yu Luo, Shuohui Yang, Jiang Lin, Dongmei Gao, Yan Zhao, Jinguo Liu, Xiangyang Shi, Xiaolin Wang

**Affiliations:** 1 Shanghai Institute of Medical Imaging, Department of Interventional Radiology, Zhongshan Hospital, Fudan University, Shanghai 200032, P. R. China; 2 College of Chemistry, Chemical Engineering and Biotechnology, Donghua University, Shanghai 201620, P. R. China; 3 Shanghai Institute of Medical Imaging, Department of Radiology, Zhongshan Hospital, Fudan University, Shanghai 200032, P. R. China; 4 Liver Cancer Institute, Zhongshan Hospital, Fudan University, Shanghai 200032, P. R. China; 5 Department of Pulmonary Medicine, Zhongshan Hospital, Fudan University, Shanghai 200032, P. R. China

## Abstract

Hepatocellular carcinoma (HCC) is the most common malignant tumor of the liver. The early and effective diagnosis has always been desired. Herein, we present the preparation and characterization of hyaluronic acid (HA)-modified, multifunctional nanoparticles (NPs) targeting CD44 receptor-expressing cancer cells for computed tomography (CT)/magnetic resonance (MR) dual-mode imaging. We first modified amine-terminated generation 5 poly(amidoamine) dendrimers (G5.NH_2_) with an Mn chelator, 1,4,7,10-tetraazacyclododecane-1,4,7,10-tetraacetic acid (DOTA), fluorescein isothiocyanate (FI), and HA. Then, gold nanoparticles (AuNPs) were entrapped within the above raw product, denoted as G5.NH_2_-FI-DOTA-HA. The designed multifunctional NPs were formed after further Mn chelation and purification and were denoted as {(Au^0^)_100_G5.NH_2_-FI-DOTA(Mn)-HA}. These NPs were characterized *via* several different techniques. We found that the {(Au^0^)_100_G5.NH_2_-FI-DOTA(Mn)-HA} NPs exhibited good water dispersibility, stability under different conditions, and cytocompatibility within a given concentration range. Because both AuNPs and Mn were present in the product, {(Au^0^)_100_G5.NH_2_-FI-DOTA(Mn)-HA} displayed a high X-ray attenuation intensity and favorable *r*_1_ relaxivity, which are advantageous properties for targeted CT/MR dual-mode imaging. This approach was used to image HCC cells *in vitro* and orthotopically transplanted HCC tumors in a unique *in vivo* model through the CD44 receptor-mediated endocytosis pathway. This work introduces a novel strategy for preparing multifunctional NPs *via* dendrimer nanotechnology.

Hepatocellular carcinoma (HCC) is the most common malignant tumor of the liver and has the sixth highest incidence rate and the second highest mortality rate of malignant tumors worldwide^1^. Nevertheless, as this condition is difficult to diagnose early, it is often treated late, which leads to low survival rates^2^,^3^. Rapid developments in molecular imaging technology over the last decade have allowed the collection of highly sensitive and specific information regarding physiological and pathological processes for the accurate diagnosis of disease^4^,^5^,^6^,^7^. Among current clinical imaging techniques, computed tomography (CT) is considered to be one of the most convenient because of its wide availability, speed, efficiency, and inexpensiveness^8^,^9^,^10^,^11^. CT is able to present images with high spatial and density resolution, and this technique can provide valuable tomographic information regarding anatomical structures^12^,^13^,^14^ and functional information^15^,^16^,^17^ through the use of high-resolution 3-dimensional (3D) technology^12^,^18^,^19^. Meanwhile, magnetic resonance (MR) imaging is one of the most powerful noninvasive medical imaging techniques with good spatial resolution and high sensitivity^20^,^21^, which can provide superior anatomical detail and tomographic information, especially in soft tissues^22^,^23^.

However, due to the limitations of individual techniques, only combinations of different techniques can provide more accurate and comprehensive diagnostic data^24^,^25^,^26^,^27^,^28^. Therefore, it is essential to develop various contrast agents for dual- or multimode imaging applications^26^,^27^,^28^. Conventional, small-molecule contrast agents have serious shortcomings, such as a lack of specificity, short half-life, and risk of nephrotoxicity at relatively high concentrations. Fortunately, superior nanoparticles (NPs) have recently been developed, including NPs with the ability to specifically target tumor cells^9^,^26^,^27^,^28^,^29^,^30^,^31^,^32^.

Previous studies have described the synthesis of different types of CT/MR dual-mode imaging contrast agents^26^,^27^,^28^. Despite the many advantages of iron as a contrast agent for T_2_-negative MR imaging^28^, T_1_ contrast agents consisting of gadolinium (Gd)/chelator complexes are the commercially available MR contrast agents used in clinical settings^33^,^34^. However, Gd ions can become highly toxic if they dissociate from NPs or complexes *in vivo*, thus resulting in severe renal damage, e.g., nephrogenic systemic fibrosis^35^. Due to the safe excretion of intracellular manganese (Mn), cytotoxicity issues related to transmetallation and bioretention are considered nominal provided that Mn remains chelated in the bolus/vascular phase^36^. In addition, as Mn ions are highly magnetized, Mn is a compatible material for MR imaging^37^. Different molecular probes have incorporated Mn to facilitate the specific delivery of probes to cancer cells and achieve targeted molecular imaging *via* MR^38^,^39^,^40^,^41^. Meanwhile, several reports have described the application of NPs in CT/MR dual-mode imaging to evaluate subcutaneous transplanted tumors *in vivo*^26^,^27^,^28^. The orthotopic tumor transplantation model is known to represent physiological and pathological processes better than the subcutaneous tumor transplantation model. Hence, we aimed to synthesize a novel NP-based CT/T_1_ MR imaging contrast agent for *in vivo* use in the orthotopic HCC model.

In some cancers, CD44 is a major cell surface marker of progression and metastasis^42^. Certain established noninvasive cell lines and normal cell types do not express CD44 receptor^43^,^44^. Metastatic cancer growth is closely related to interactions between the tumor stroma and the microenvironment involving CD44^43^,^44^. Moreover, hyaluronic acid (HA) is a molecule that can specifically bind to CD44 receptors in a highly efficient manner^45^. This important characteristic of HA, as well as its biocompatibility, biodegradability, and easily modified structure, makes HA-based materials extremely attractive for applications such as the tumor-targeted delivery of imaging agents and the diagnosis and treatment of cancer^46^,^47^,^48^,^49^,^50^.

In this study, 2,2′,2″-(10-(2-(2,5-dioxopyrrolidin-1-yloxy)-2-oxoethyl)-1,4,7,10-tetraazacyclododecane-1,4,7-triyl) triacetic acid (DOTA-NHS) and fluorescein isothiocyanate (FI) were conjugated to the surface of G5.NH_2_ dendrimers, and targeting HA ligands were also coupled onto the dendrimer surface *via* 1-ethyl-3-[3-dimethylaminopropyl] carbodiimide hydrochloride (EDC) coupling chemistry. Then, the product (i.e., G5.NH_2_-FI-DOTA-HA) was used as a template to entrap AuNPs and chelate Mn. HA-targeted Mn-loaded dendrimer-entrapped AuNPs (i.e., {(Au^0^)_100_G5.NH_2_-FI-DOTA(Mn)-HA}) were formed (Fig. 1) without acetylating the remaining terminal amines of dendrimers. The developed {(Au^0^)_100_G5.NH_2_-FI -DOTA(Mn)-HA} NPs were characterized *via*^1^H nuclear magnetic resonance (NMR) spectroscopy, dynamic light scattering (DLS), transmission electron microscopy (TEM), and inductively coupled plasma-atomic emission spectroscopy (ICP-AES). The cytocompatibility of the NPs was evaluated using a cytotoxicity assay and cell morphology observations. Furthermore, the formed {(Au^0^)_100_G5.NH_2_-FI-DOTA (Mn)-HA} NPs were applied in the CT/MR dual-mode imaging of HCCLM3 cells *in vitro* and orthotopically transplanted HCC tumors *in vivo*.

## Experimental

### Materials

Ethylenediamine core amine-terminated G5.NH_2_ poly(amidoamine) dendrimers with a polydispersity index (PDI) less than 1.08 were purchased from Dendritech (Midland, MI). DOTA-NHS was purchased from CheMatech (Dijon, France). HA with molecular weight of 6 k Da, sodium hydroxide, EDC, and N-hydroxysuccinimide (NHS) were purchased from J&K Chemical Ltd. (Shanghai, China). HCCLM3 cells (an HCC cell line) were obtained from the Liver Cancer Institute, Zhongshan Hospital, Fudan University (Shanghai, China). Fetal bovine serum (FBS), streptomycin, penicillin, and Dulbecco’s modified Eagle medium (DMEM) were obtained from Hangzhou Jinuo Biomedical Technology (Hangzhou, China). Triethylamine, acetic anhydride, HAuCl_4_·4H_2_O, and all other chemicals and solvents were purchased from Sinopharm Chemical Reagent Co. Ltd. (Shanghai, China). The water used in all experiments was purified by a Milli-Q Plus 185 water purification system (Millipore, Bedford, MA) with a resistivity greater than 18 MΩ cm. Regenerated cellulose dialysis membranes (MWCO = 14,000 or 1,000) were acquired from Thermo Fisher Scientific (Pittsburgh, PA).

### Synthesis of {(Au^0^)_100_G5.NH_2_-FI-DOTA(Mn)-HA} NPs

In all, 35 molar equivalents of DOTA-NHS (26.25 mg, 35 μmol) dissolved in DMSO (10 mL) were added dropwise into a DMSO solution of G5.NH_2_ dendrimers (26 mg, 1 μmol, 10 mL), forming the raw G5.NH_2_-DOTA product after vigorous magnetic stirring for 1 d. Then, 20 molar equivalents of HA (120.49 mg, 20 μmol) preactivated by NHS (11.91 mg, 100 μmol, in 1 mL of DMSO) and EDC (19.11 mg, 100 μmol, in 1 mL of DMSO) were added dropwise into the aforementioned G5.NH_2_-DOTA solution to form the raw G5.NH_2_-DOTA-HA product after vigorous magnetic stirring for 3 d. FI (1.91 mg, 5 μmol, in 1 mL of DMSO) was then conjugated to G5.NH_2_-DOTA-HA to form the raw G5.NH_2_-FI-DOTA-HA product after vigorous magnetic stirring for 1 d.

The procedure used to synthesize {(Au^0^)_100_G5.NH_2_-FI-DOTA(Mn)-HA} was similar to what has been previously reported, with slight modifications^26^,^27^. In brief, 100 molar equivalents of HAuCl_4_.4H_2_O in an aqueous solution (30 mg/mL, 2.78 mL) were added into the G5.NH_2_-FI-DOTA-HA aqueous solution mentioned above under vigorous stirring for 30 min. An ice-cold aqueous NaBH_4_ solution (18.92 mg, 0.5 mmol, in 1 mL of H_2_O) with a 5-fold molar excess of Au salt was added into the aforementioned solution under vigorous stirring for 2 h. Then, 70 molar equivalents of MnSO_4_ in an aqueous solution (13.52 mg, in 1 mL of H_2_O) were added dropwise into the aforementioned G5 dendrimer solution under vigorous stirring for 24 h to synthesize the targeted {(Au^0^)_100_G5.NH_2_-FI-DOTA(Mn)-HA} NPs. Extensive dialysis was applied to remove the excess reactants and by-products in the reaction mixture, and subsequent lyophilization was used to obtain the final product.

The small positive surface charge of the {(Au^0^)_100_G5.NH_2_-FI-DOTA(Mn)-HA} NPs allowed the commonly used acetylation step to be omitted. In the control group, the CD44 receptors were blocked with free HA at a concentration of 25 mM for half an hour before the targeted NPs were injected *via* the tail vein.

### Characterization techniques

DLS and zeta potential measurements were conducted using a Malvern Zetasizer Nano ZS model ZEN3600 (Worcestershire, U.K.) with a standard 633-nm laser. Prior to taking the measurements, the samples were dissolved in water (0.1 mg/mL). We used TEM (JEOL 2010F, Tokyo, Japan) with an accelerating voltage of 200 kV to characterize the morphology of the {(Au^0^)_100_G5.NH_2_-FI-DOTA(Mn)-HA} NPs. TEM samples were prepared by depositing a dilute NP suspension (6 μL) onto carbon-coated copper grids and allowing it to air-dry before observation. The TEM image selections were random, and the average size and size distribution of more than 300 NPs were recorded using ImageJ software. ICP-AES (Leeman Prodigy, USA) was employed to analyze the composition of Au and Mn within the multifunctional NPs. A GE LightSpeed VCT imaging system (GE Medical Systems) was used for CT scanning at 80 mA, 100 kV, and a slice thickness of 0.625 mm. Solutions of {(Au^0^)_100_G5.NH_2_-FI-DOTA(Mn)-HA} NPs (0.2 mL) with different concentrations of Au were prepared in 2.0-mL Eppendorf tubes, and the CT value (Hounsfield units, HU) of each sample was measured. The concentration of Au in each sample ranged from 0.01 to 0.12 mM. T_1_ relaxometry of {(Au^0^)_100_G5.NH_2_-FI-DOTA(Mn)-HA} was performed using a 0.5-T Mini MR system (Niumeg, Shanghai, China). Solutions of {(Au^0^)_100_G5.NH_2_-FI-DOTA(Mn)-HA} NPs (1 mL) with different concentrations of Mn were prepared in 2-mL Eppendorf tubes by diluting the NP suspensions with water containing an Mn concentration ranging from 0.2 to 1.0 mM. The following system parameters were used: repetition time (TR) = 500 ms; echo time (TE) = 25 ms; slice thickness = 2 mm; matrix = 256 × 256; field of view (FOV) = 120 mm; and excitation number = 1. T_1_ relaxivity (r_1_) was determined by linearly fitting 1/T_1_ (s^−1^) as a function of the Mn concentration (mM).

### Cytotoxicity assay and cell morphology observations

An MTT assay was applied to measure the *in vitro* cytotoxicity of the {(Au^0^)_100_G5.NH_2_-FI-DOTA(Mn)-HA} NPs in a routine culture environment with 10% FBS (heat-inactivated, 1% penicillin-streptomycin) and 5% CO_2_ at 37 °C.

HCCLM3 cells suspended in medium were seeded in a 96-well plate at a density of 1 × 10^4^ cells/well with 200 μL per well and were incubated overnight. NPs with an Mn concentration ranging from 0–100 μg/mL were added into each well, and the cells were incubated for an additional 24 h. The mixture was then carefully removed, and the cells were washed twice with phosphate-buffered saline (PBS). Then, 20 μL of MTT solution (5 mg/mL in PBS) was added into each well, and the cells were cultured for another 4 h at 37 °C and 5% CO_2_. To dissolve the insoluble formazan crystals, the medium was carefully discarded and replaced with 200 μL of DMSO. Finally, the absorbance of each sample was measured at 570 nm using a Thermo Fisher Scientific Multiskan MK3 ELISA reader (Thermo Fisher Scientific, Hudson, NH).

The morphology of the HCCLM3 cells was observed to evaluate the cytotoxicity of the {(Au^0^)_100_G5.NH_2_-FI-DOTA(Mn)-HA} NPs after the cells were treated with the NPs at Mn concentrations of 0, 10, 20, 50, 75, and 100 μg/mL for 24 h. A Leica DM IL LED inverted phase-contrast microscope was employed to observe the cell morphology of each sample at a magnification of 200×.

### *In vitro* cellular uptake assay

The cellular uptake of the {(Au^0^)_100_G5.NH_2_-FI-DOTA(Mn)-HA} NPs *in vitro* was assessed by flow cytometry. HCCLM3 cells^51^, which highly express CD44, were used for this experiment. Cells were seeded into each well of a 12-well plate at a density of 2 × 10^5^ cells/well and incubated with 2 mL of DMEM overnight. NPs with Mn concentrations of 10, 25, 75, and 100 μg/mL were added into the wells, and the cells were incubated for an additional 4 h. Cells treated with PBS and cells treated with HA followed by NPs were used as controls.

### *In vivo* CT/MR imaging

All animal experiments were approved by the ethical committee of Zhongshan Hospital. A subcutaneous HCCLM3 tumor transplantation model was established in 6-week-old BALB/c nu/nu male mice (SLAC, Shanghai, China) *via* the subcutaneous injection of HCCLM3 cells (5 × 10^6^ cells in 150 μL of PBS). When the maximum diameter of the subcutaneous tumors reached 8 mm, the tumor-bearing mice were euthanized. Then, each subcutaneous tumor was removed surgically, and the non-necrotic tumor tissue was cut into small pieces (1 mm^3^) for further orthotopic implantation into the livers of a different set of 6-week-old male mice, thereby establishing the orthotopic HCCLM3 tumor model.

During the operation, the mice were anesthetized by an intraperitoneal injection of pentobarbital sodium (40 mg/kg). Three weeks after the orthotopic tumor implantation, the mice underwent CT/MR scanning. Then, a solution of {(Au^0^)_100_G5.NH_2_-FI-DOTA(Mn)-HA} NPs (0.3 mL, [Au] = 120 mM) in PBS was administered to the mice *via* a tail vein injection. CT scans were performed at 0 min, 30 min, 1 h, and 2 h post-injection using a GE LightSpeed VCT system. A GE Advantage Workstation 4.5 (AW4.5) was used for image reconstruction. MR scanning was performed at 0 min, 30 min, 1 h, and 2 h post-injection using a 3.0-T Magnetom Verio MR system with a wrist receiver coil and the following parameters: TR/TE = 582/24 ms; FOV = 120 mm; FOV phase = 100%; matrix = 416 × 416; and slice thickness = 1.5 mm. Three mice were included in each group.

### *In vivo* biodistribution of {(Au^0^)_100_G5.NH_2_-FI-DOTA(Mn)-HA} NPs

The tumor-bearing mice were used to study the *in vivo* biodistribution of the {(Au^0^)_100_G5.NH_2_-FI-DOTA(Mn)-HA} NPs. The mice were first anesthetized by an intraperitoneal injection of pentobarbital sodium (40 mg/kg). Then, {(Au^0^)_100_G5.NH_2_-FI-DOTA(Mn)-HA} NPs (0.3 mL in PBS solution, [Au] = 120 mM) were injected *via* the tail vein; the mice were euthanized at 24 h post-injection. The major organs, i.e., liver, spleen, kidneys, lungs, and heart, were surgically removed along with the tumor and weighed. The tissues were cut into small pieces (1-2 mm^3^) for aqua regia digestion (nitric acid:hydrochloric acid = 1:3 (v/v)). The Au content was measured by ICP-AES.

### Statistical analysis

The experimental data were analyzed by a single-factor analysis (one-way ANOVA). The results were considered significant at *P* < 0.05; (*), (**), and (***) were used to indicate *P* < 0.05, *P* < 0.01, and *P* < 0.001, respectively.

## Results and Discussion

### Synthesis and characterization of {(Au^0^)_100_G5.NH_2_-FI-DOTA(Mn)-HA} NPs

**Figure 1:**
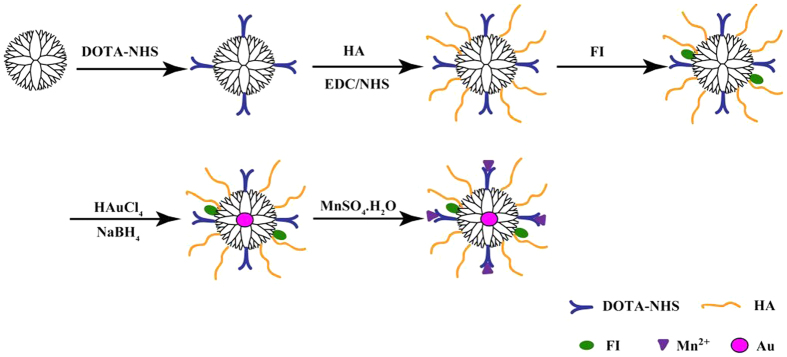
Schematic representation of the synthesis of the {(Au^0^)_100_G5.NH_2_-FI-DOTA(Mn)-HA} NPs.

In this study, {(Au^0^)_100_G5.NH_2_-FI-DOTA(Mn)-HA} NPs were formed using an NaBH_4_ reduction route, as previously reported^26^,^27^. Both AuNPs and Mn were integrated into one multifunctional NP platform for CT/MR dual-mode imaging applications. First, DOTA-NHS was covalently linked to the surface of G5.NH_2_ dendrimers. Then, HA and FI were coupled to the dendrimers separately. AuNPs were then entrapped by G5.NH_2_-DOTA-FI-HA *via* sodium borohydride reduction chemistry. Following AuNP entrapment, Mn ions were chelated to the NPs, producing multifunctional {(Au^0^)_100_G5.NH_2_-FI-DOTA(Mn)-HA} NPs suitable for both CT and MR imaging (Fig. 1).

Our NMR results (Figure S1a), which were obtained using a previously reported method^27^, indicated that each G5 dendrimer was linked with approximately 23.6 DOTA moieties. These linked DOTA moieties can be used to chelate Mn for T_1_ MR imaging. Based on the feeding ratio, the theoretical number of DOTA moieties linked to each G5 dendrimer was 30, but the actual number was slightly smaller. We utilized the same method to calculate that approximately 13.6 HA and 3.7 FI moieties were linked to each G5 dendrimer (Figure S1b,c).

The zeta potential and hydrodynamic size of the {(Au^0^)_100_G5.NH_2_-FI-DOTA(Mn)-HA} NPs were recorded (Table 1). The final product clearly exhibited a slight positive charge (+6.1 mV) due to the HA coupled to the dendrimer terminal amines and the omission of acetylating the remaining dendrimer terminal amines^26^,^27^. The small amount of positive charge on the NP surface may have been caused by some of the G5 amines being used to stabilize the AuNPs. DLS was used to assess the hydrodynamic size of the developed NPs dissolved in water (Table 1). The {(Au^0^)_100_G5.NH_2_-FI-DOTA(Mn)-HA} NPs clearly displayed a hydrodynamic size of 245.3 nm. The NPs exhibited both an acceptable PDI and excellent colloidal stability.

**Figure 2:**
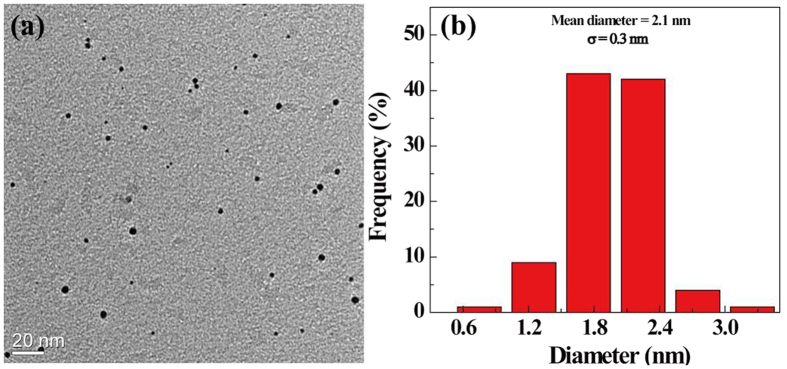
TEM image (**a**) and size distribution histograms (**b**) of the {(Au^0^)_100_G5.NH_2_-FI-DOTA(Mn)-HA} NPs. The scale bar in each panel represents 20 nm.

The morphology and size of the {(Au^0^)_100_G5.NH_2_-FI-DOTA(Mn)-HA} NPs were observed by TEM (Fig. 2). The formed NPs were nearly spherical in shape (Fig. 2a) and had a mean diameter of 2.1 nm with a relatively uniform size distribution (Fig. 2b). The size measured by DLS was larger than that measured by TEM, likely because DLS characterizes the size of {(Au^0^)_100_G5.NH_2_-FI-DOTA(Mn)-HA} NP clusters in an aqueous solution, whereas TEM reveals the size of individual AuNPs^26^,^27^,^28^.

### Stability

The stability of these NPs is essential for their biomedical application. To evaluate the stability of the prepared {(Au^0^)_100_G5.NH_2_-FI-DOTA(Mn)-HA} NPs, the NPs were dissolved in different solutions (i.e., water, PBS, or cell culture medium). After the NPs were stored for one month at room temperature, no precipitate was observed (Figure S2). Meanwhile, as described in the literature^28^, we also measured the hydrodynamic size of the NPs after 7 days of storage at room temperature. The hydrodynamic diameter of the NPs was 266.5 nm, which was not significantly different from the value observed before storage.

### T_1_ relaxometry

**Figure 3:**
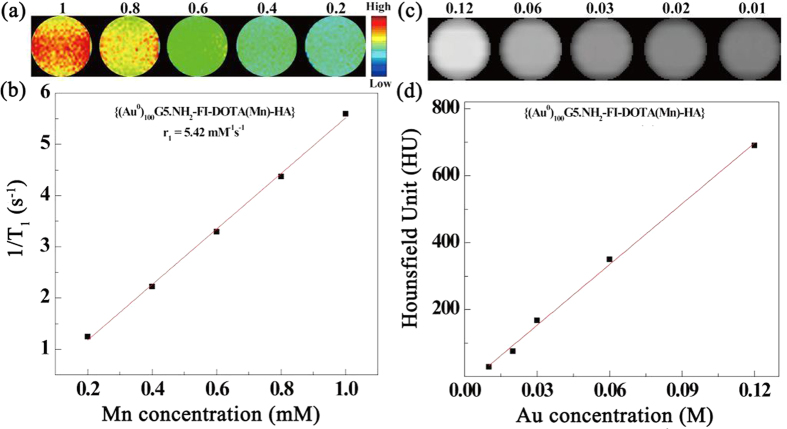
T_1_-weighted MR imaging (**a**) and linear fitting of 1/T_1_ (**b**) of the {(Au^0^)_100_G5.NH_2_-FI-DOTA(Mn)-HA} NPs at Mn concentrations of 0.2, 0.4, 0.6, 0.8, and 1 mM. CT phantom images (**c**) and X-ray attenuation (HU) (**d**) of the {(Au^0^)_100_G5.NH_2_-FI-DOTA(Mn)-HA} NPs as a function of Au concentration (0.01, 0.02, 0.03, 0.06, and 0.12 mM).

The paramagnetic Mn complexes render the NPs suitable for MR imaging. A 0.5-T Mini MR system (Niumeg, Shanghai, China) was used to calculate the longitudinal relaxation time (T_1_) of the {(Au^0^)_100_G5.NH_2_-FI-DOTA(Mn)-HA} NPs by measuring the MR signal intensity of aqueous NP solutions with different Mn concentrations (i.e., 0.2, 0.4, 0.6, 0.8, and 1 mM). The T_1_-weighted MR images (Fig. 3a) clearly show that the MR signal intensity increases with increasing Mn concentration. By linearly fitting the relaxation rate (1/T_1_) *versus* the Mn concentration, the r_1_ relaxivity of the {(Au^0^)_100_G5.NH_2_-FI-DOTA(Mn)-HA} NPs was calculated to be 5.42 mM^−1^s^−1^ (Fig. 3b).

### X-ray attenuation measurements

The {(Au^0^)_100_G5.NH_2_-FI-DOTA(Mn)-HA} NPs could be used for CT imaging because of presence of the AuNPs. As previously reported by Cao *et al*.^52^, Au is superior to iodine in terms of X-ray attenuation (e.g., Omnipaque) because Au has a higher atomic number. Therefore, AuNPs have been widely applied in CT contrast agents. The results clearly show that the CT value increases as the Au concentration increases (Fig. 3c,d). By linearly fitting the attenuation intensity *versus* the Au concentration, a dose-dependent relation was obtained.

### *In vitro* cytotoxicity

**Figure 4:**
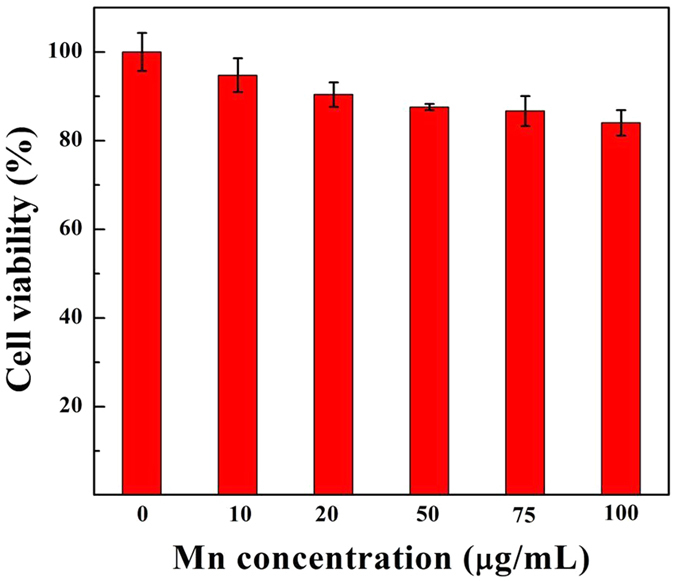
MTT assay results of HCCLM3 cell viability after treatment for 24 h with {(Au^0^)_100_G5.NH_2_-FI-DOTA(Mn)-HA} NPs at Mn concentrations of 10, 20, 50, 75, and 100 μg/mL; PBS was used as a blank control.

Prior to biomedical application, the cytocompatibility of the developed {(Au^0^)_100_G5.NH_2_-FI-DOTA(Mn)-HA} NPs was evaluated using an MTT colorimetric assay (Fig. 4). After 24 h of incubation with HCCLM3 cells, no significant cytotoxicity from the {(Au^0^)_100_G5.NH_2_-FI-DOTA(Mn)-HA} NPs was observed at any of the tested Mn concentrations. Compared to the PBS control, no significant difference (*P* > 0.05) in HCCLM3 cell viability was observed even at the highest Mn concentration (100 μg/mL). Cell viability remained at more than 80%. Clearly, the produced NPs are cytocompatible in the given Mn concentration range.

To further evaluate NP cytotoxicity, after the HCCLM3 cells were treated with {(Au^0^)_100_G5.NH_2_-FI-DOTA(Mn)-HA} NPs at Mn concentrations of 10, 20, 50, 75, and 100 μg/mL for 24 h, the morphology of the cells was then observed by phase-contrast microscopy (Figure S3). The results showed that the morphology of the HCCLM3 cells treated with the {(Au^0^)_100_G5.NH_2_-FI-DOTA(Mn)-HA} NPs at Mn concentrations of 10–100 μg/mL (Figure S3b–f) was similar to that of the cells in the control group (treated with PBS) (Figure S3a). The MTT data are supported by the cell morphology results, thereby validating the cytocompatibility of the {(Au^0^)_100_G5.NH_2_-FI-DOTA(Mn)-HA} NPs and their suitability for *in vivo* CT/MR imaging applications.

### Flow cytometry assay

The NPs were modified by FI molecules and could be analyzed by flow cytometry through the binding of the {(Au^0^)_100_G5.NH_2_-FI-DOTA(Mn)-HA} NPs to the target cells. Cells with blocked CD44 receptors were used as a control. At different Mn concentrations, the fluorescence intensity of HCCLM3 cells with unblocked CD44 receptors was much stronger than that of cells with blocked CD44 receptors (Figures S4 and 5). The enhanced cellular uptake of the {(Au^0^)_100_G5.NH_2_-FI-DOTA(Mn)-HA} NPs should be related to the modified HA molecules that can specifically target HCCLM3 cells *via* the CD44 receptor-mediated pathway.

### *In vivo* targeted CT/MR imaging of orthotopic HCC tumors

**Figure 6:**
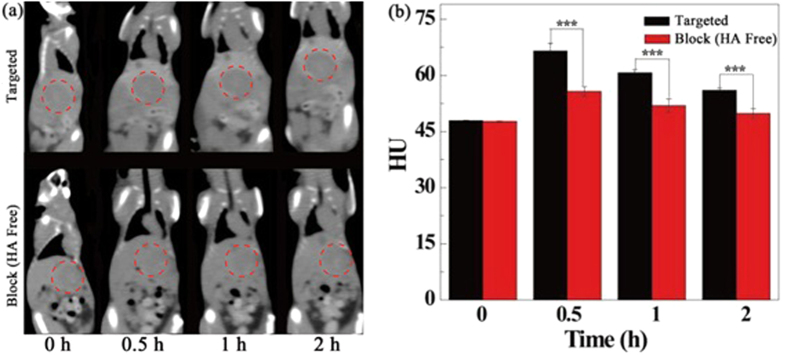
*In vivo* CT images of orthotopic liver tumors at different times after a 0.3-mL intravenous injection of a {(Au^0^)_100_G5.NH_2_-FI-DOTA(Mn)-HA} NP solution (0.3 mL in PBS, [Au] = 120 mM).

**Figure 7:**
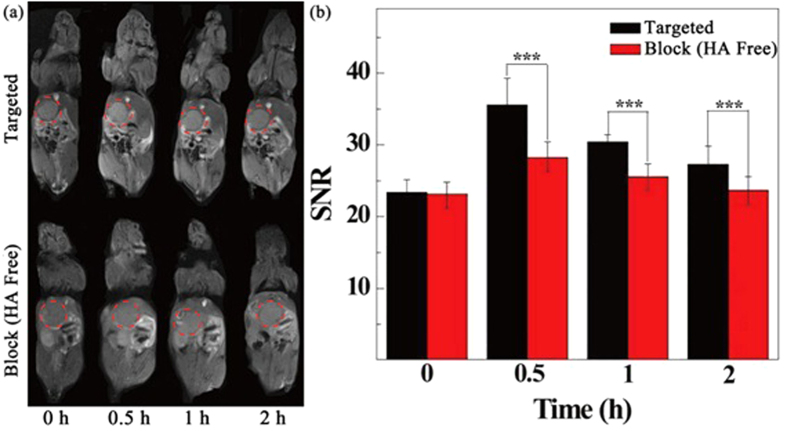
*In vivo* MR images of orthotopic liver tumors at different times after an intravenous injection of 0.3 mL of a {(Au^0^)_100_G5.NH_2_-FI-DOTA(Mn)-HA} NP (300 μg Mn) solution in PBS.

**Figure 8:**
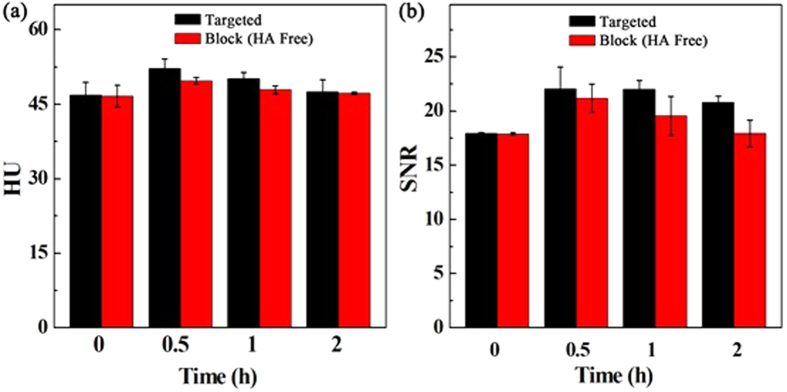
*In vivo* CT images of normal liver tissue at different times after a 0.3-mL intravenous injection of a {(Au^0^)_100_G5.NH_2_-FI-DOTA(Mn)-HA} NP solution (0.3 mL in PBS, [Au] = 120 mM) (**a**). *In vivo* MR images of normal liver tissue at different times after an intravenous injection of 0.3 mL of a {(Au^0^)_100_G5.NH_2_-FI-DOTA(Mn)-HA} NP (300 μg Mn) (**b**) solution in PBS.

The {(Au^0^)_100_G5.NH_2_-FI-DOTA(Mn)-HA} NPs were next employed to verify the feasibility of targeted CT/MR imaging of tumors *in vivo* (Figs 6 and 7). CD44 receptor blocking was used for the control. Figures 6 and 7 show the contrast enhancement in the CT and T_1_-weighted MR images of tumors at different time points after the tail vein injection of the {(Au^0^)_100_G5.NH_2_-FI-DOTA(Mn)-HA} and free HA + {(Au^0^)_100_G5.NH_2_-FI-DOTA (Mn)-HA} NPs. The CT and MR signal intensity values of the tumors appeared to reach their maxima at 0.5 h post-injection. In the experimental group, this should be attributable to the fact that the NPs accumulate in the tumor region *via* the enhanced permeability and retention (EPR) effect as well as active targeting. However, in the control group, this effect is likely only attributable to NP accumulation *via* the EPR effect because of the blocked CD44 receptors. Based on the quantitative changes in the CT and MR signal intensity values over time after the actively targeted NPs were injected, the tumor CT density and MR signal intensity values are highest at 0.5 h post-injection and partly recover at 1 h post-injection. The CT and MR signal intensity values of the tumors treated with the NPs in the experimental group were obviously higher than those of the free HA in the control group at the same time point (*P* < 0.001). At 1 h post-injection with free HA + NPs, the NPs began to be metabolized and leave the tumor site, which led to the recovery of the CT value and MR signal, precisely the opposite of the significantly higher CT density and MR signal intensity values maintained after the NP injection without free HA. The CT/MR imaging results of the orthotopic tumors showed that the synthesized {(Au^0^)_100_G5.NH_2_-FI-DOTA(Mn)-HA} NPs can be used as a nanoprobe for effective, targeted CT/MR dual-mode imaging *in vivo.* Our results suggest that {(Au^0^)_100_G5.NH_2_-FI-DOTA(Mn)-HA} NPs have the ability to target cancer cells through an HA-mediated targeting pathway because the targeting ability was weakened when CD44 receptors were blocked. Meanwhile, we also measured the CT and MR signal intensity values of normal livers, and no significant differences were found between the two groups at the different time points (Fig. 8). These results also clearly showed that the CT density and MR signal intensity values were greater in the tumor tissue than in the normal liver tissue. The CT/MR images of orthotopic HCC *in vivo* could be used to easily distinguish tumor tissue from normal tissue.

### *In vivo* biodistribution

It is crucial to know the biodistribution of the synthesized {(Au^0^)_100_G5.NH_2_-FI-DOTA(Mn)-HA} NPs prior to their application in advanced *in vivo* biomedical imaging. The *in vivo* biodistribution of the {(Au^0^)_100_G5.NH_2_-FI-DOTA(Mn)-HA} NPs in major organs, such as the liver, spleen, kidneys, lungs, and heart, as well as in the tumor, was analyzed by ICP-AES at 24 h post-injection (Fig. 8). After the injection of both NP samples, the Au concentration in the measured organs was obviously higher in the treatment group than in the blank control group. The main uptake of Au occurred in the liver, spleen and lungs in the treatment group, while relatively little uptake occurred in the other tissues (i.e., heart, kidneys, and tumor). The biodistribution data of the Au indicated that the particles could be delivered to the tumor tissue after escaping the reticuloendothelial system. The targeting property of these {(Au^0^)_100_G5.NH_2_-FI-DOTA(Mn)-HA} NPs was clearly demonstrated, as the Au concentration in the tumor region of the mice treated with the targeted {(Au^0^)_100_G5.NH_2_-FI-DOTA(Mn)-HA} NPs was double that in mice treated with the non-targeted NPs. HA plays an important role in this process by enabling the highly efficient delivery of NPs to the tumor region *in vivo* for targeted CT/MR tumor imaging.

## Conclusions

In summary, {(Au^0^)_100_G5.NH_2_-FI-DOTA(Mn)-HA} NPs with X-ray attenuation favorable for CT imaging and r_1_ relaxivity suitable for T_1_-weighted MR imaging were developed and applied in an orthotopic HCC tumor model. Dendrimers were used as a template to entrap AuNPs within and chelate Mn ions onto the template surface. EDC coupling chemistry was used to couple HA molecules onto the G5 dendrimer surfaces, thereby endowing the NPs with the ability to actively target CD44 receptor-expressing cancer cells. The favorable characteristics of these multifunctional NPs, including water solubility, colloidal stability, and biocompatibility, make them extremely attractive for potential use in the CT/MR imaging of tumors *in vivo via* an active, HA-mediated targeting pathway. With the application of this strategy, various dual- or multimode imaging contrast agents may be designed for the accurate diagnosis of various types of malignant tumors.

## Additional Information

**How to cite this article**: Wang, R. *et al*. Hyaluronic acid-modified manganese-chelated dendrimer-entrapped gold nanoparticles for the targeted CT/MR dual-mode imaging of hepatocellular carcinoma. *Sci. Rep.* **6**, 33844; doi: 10.1038/srep33844 (2016).

## Supplementary Material

Supplementary Information

## Figures and Tables

**Figure 5:**
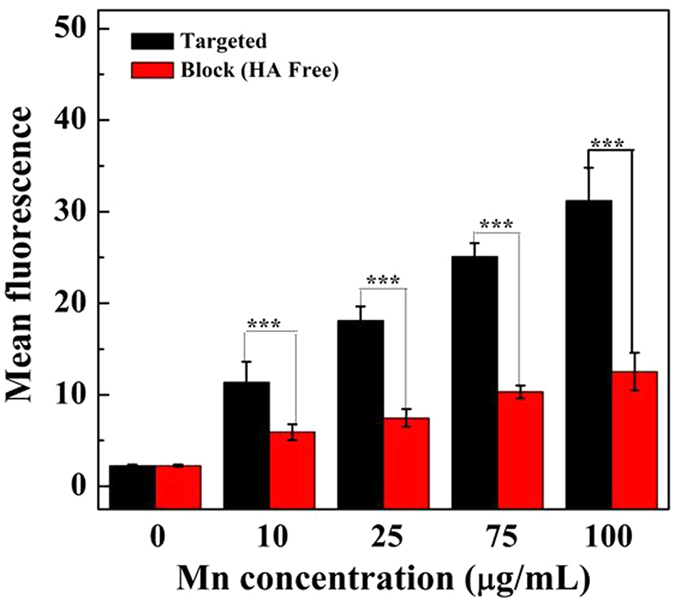
Flow cytometric results of the mean fluorescence of HCCLM3 cells treated for 4 h with {(Au^0^)_100_G5.NH_2_-FI-DOTA(Mn)-HA} NPs at Mn concentrations of 10, 25, 75, and 100 μg/mL.

**Figure 9:**
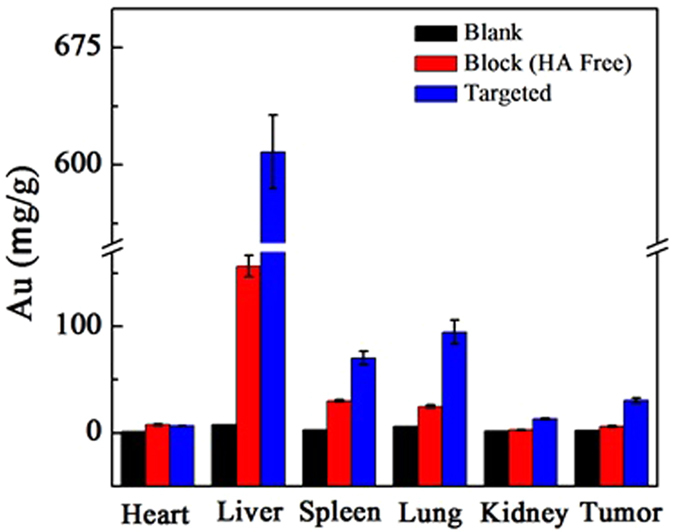
Biodistribution in the major organs of the mice, i.e., heart, liver, spleen, lungs, and kidneys, as well as in the tumors, at 24 h after the intravenous injection of a PBS solution containing {(Au^0^)_100_G5.NH_2_-FI-DOTA(Mn)-HA} NPs (0.3 mL in PBS, [Au] = 120 mM).

**Table 1:** The zeta potential and hydrodynamic size of the {(Au^0^)_100_G5.NH_2_-FI-DOTA(Mn)-HA} NPs.

Sample		G5.NH_2_-FI-DOTA-HA	{(Au^0^)_100_G5.NH_2_-FI-DOTA(Mn)}	{(Au^0^)_100_G5.NH_2_-FI-DOTA(Mn)-HA}
Zeta potential (mV)		+6.4 ± 0.6	+24.7 ± 0.3	+6.1 ± 0.7
Hydrodynamic size (nm)	Water	189.2 (PDI = 0.219)	196.5 (PDI = 0.221)	245.3 (PDI = 0.293)
	PBS	187.5 (PDI = 0.231)	190.1 (PDI = 0.245)	238.7 (PDI = 0.315)
	Medium	188.3 (PDI = 0.219)	196.6 (PDI = 0.268)	248.6 (PDI = 0.334)
