# Supplementary material for: Hyaluronic acid-modified manganese-chelated dendrimer-entrapped gold nanoparticles for the targeted CT/MR dual-mode imaging of hepatocellular carcinoma
Source: Sci Rep. 2016 Sep 22;6:33844. doi: 10.1038/srep33844 (PMC5032118; doi:10.1038/srep33844)
Supplement: Supplementary Information [file srep33844-s1.doc]

Electronic Supplementary Information

**Hyaluronic acid-modified manganese-chelated dendrimer-entrapped gold nanoparticles for the targeted CT/MR dual-mode imaging of hepatocellular carcinoma**

Ruizhi Wang ^§1^, Yu Luo ^§2^, Shuohui Yang ^3^, Jiang Lin ^3^, Dongmei Gao ^4^, Yan Zhao ^4^, Jinguo Liu ^5^, Xiangyang Shi ^2^**, Xiaolin Wang ^1^*

^1^ Shanghai Institute of Medical Imaging, Department of Interventional Radiology, Zhongshan Hospital, Fudan University, Shanghai 200032, P. R. China

^2^ College of Chemistry, Chemical Engineering and Biotechnology, Donghua University, Shanghai 201620, P. R. China

^3^ Shanghai Institute of Medical Imaging, Department of Radiology, Zhongshan Hospital, Fudan University, Shanghai 200032, P. R. China

^4^ Liver Cancer Institute, Zhongshan Hospital, Fudan University, Shanghai 200032, P. R. China

^5^ Department of Pulmonary Medicine, Zhongshan Hospital, Fudan University, Shanghai 200032, P. R. China

* Corresponding author. Tel.: + 86 21 64041990 3278; fax: + 86 21 64041990 3278.

** Corresponding author. Tel.: + 86 21 67792656 804; fax: + 86 21 67792306 804.

E-mail addresses: fduwangxiaolin@hotmail.com (X. Wang), xshi@dhu.edu.cn (X. Shi).

^§^These authors contributed equally to this work.


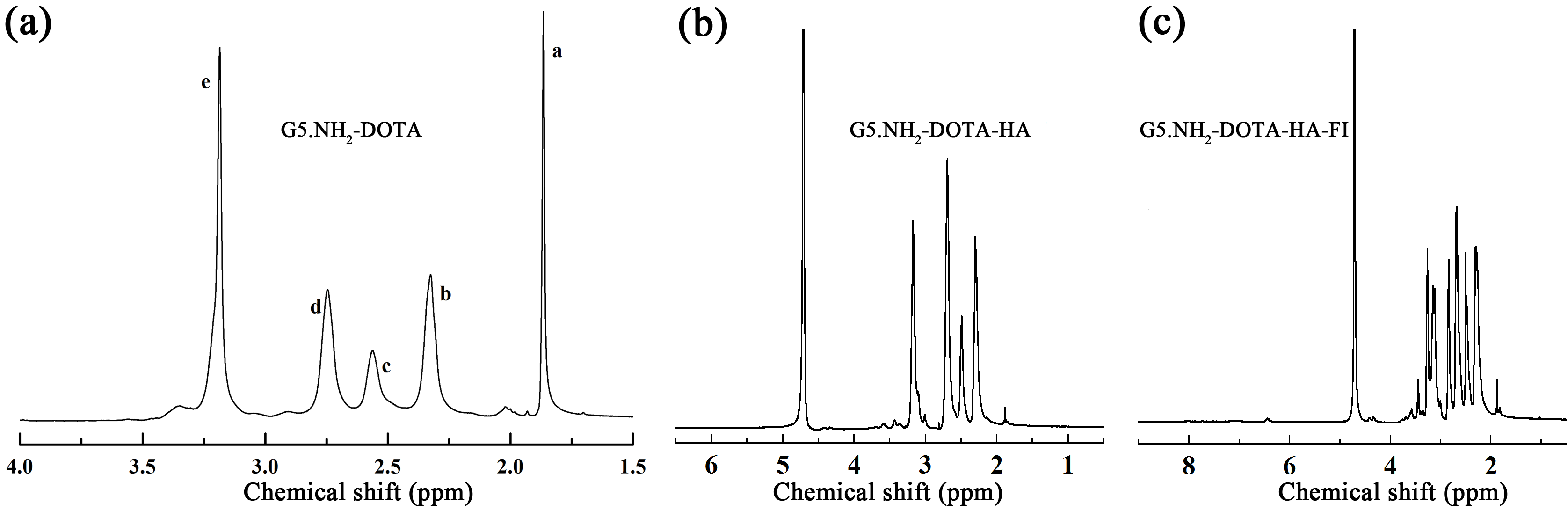


**Figure S1.** ^1^H NMR spectra of G5.NH2-DOTA (a), G5.NH2-DOTA-HA (b), and G5.NH_2_-DOTA-HA-FI (c).

**Figure S1**

**Wang et al.**

**
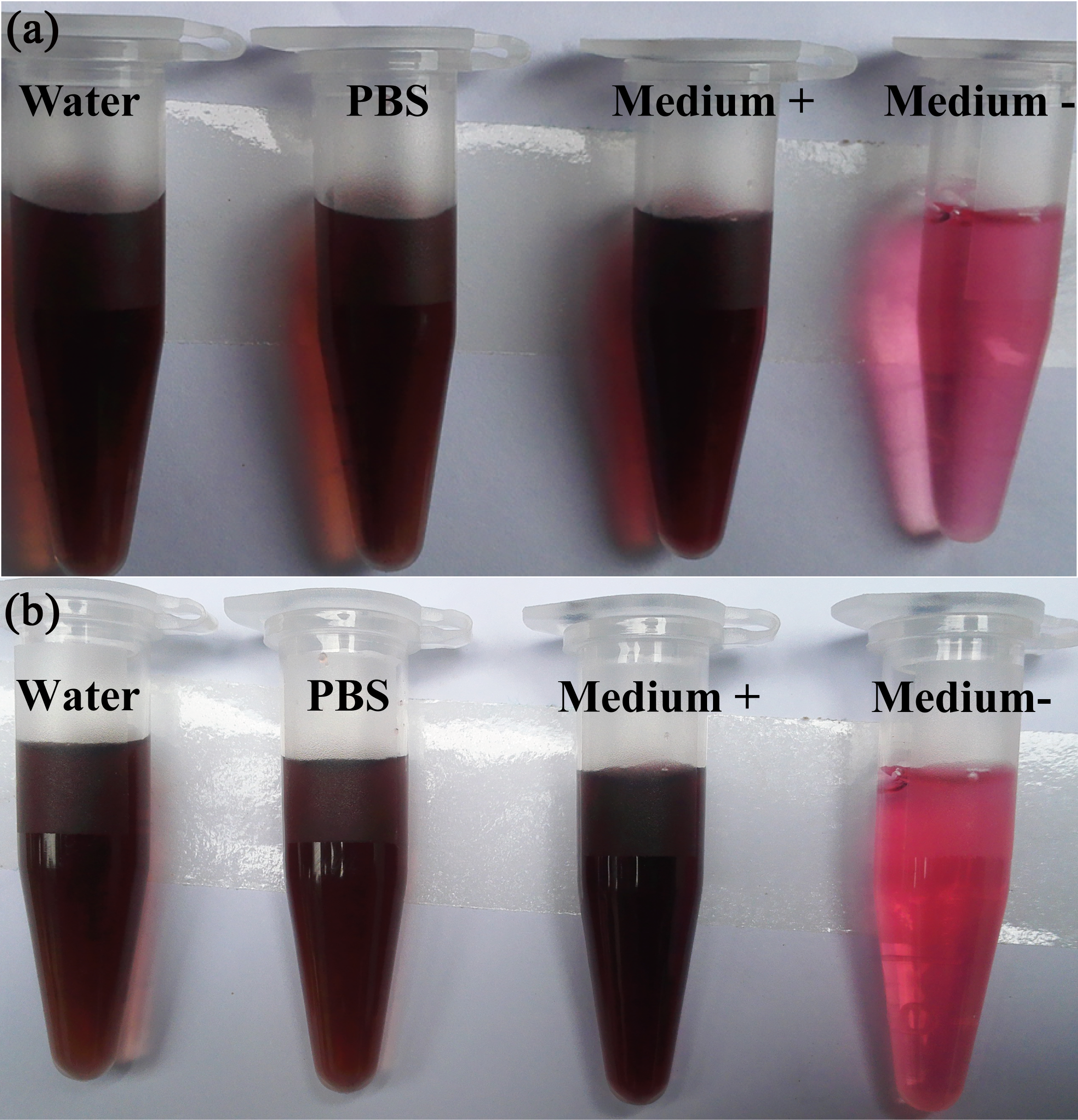
**

**Figure S2.** Images of the {(Au^0^)_100_G5.NH_2_-FI-DOTA(Mn)-HA} NPs dispersed in water, PBS, and cell culture medium (medium+), as well as pure culture medium without NPs (medium-, as a control) before (a) and after (b) one month of storage at room temperature.

**Figure S2**

**Wang et al.**

**
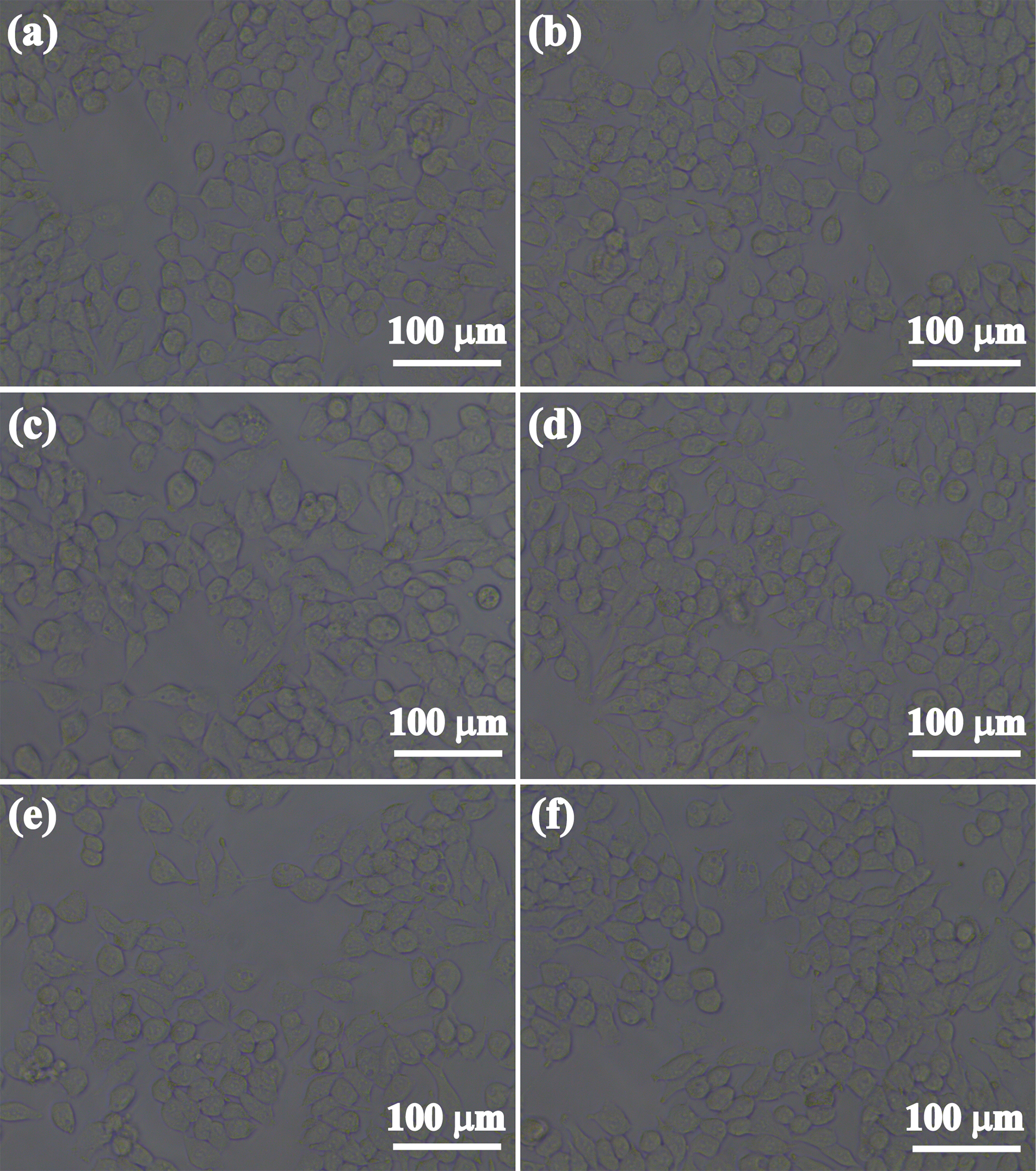
**

**Figure S3.** Phase-contrast microscopy images showing the viability of HCCLM3 cells after being treated for 24 h with PBS (a) or {(Au^0^)_100_G5.NH_2_-FI-DOTA(Mn)-HA} NPs at an Mn concentration of 10 (b), 20 (c), 50 (d), 75 (e), or 100 (f) μg/mL.

**Figure S3**

**Wang et al.**


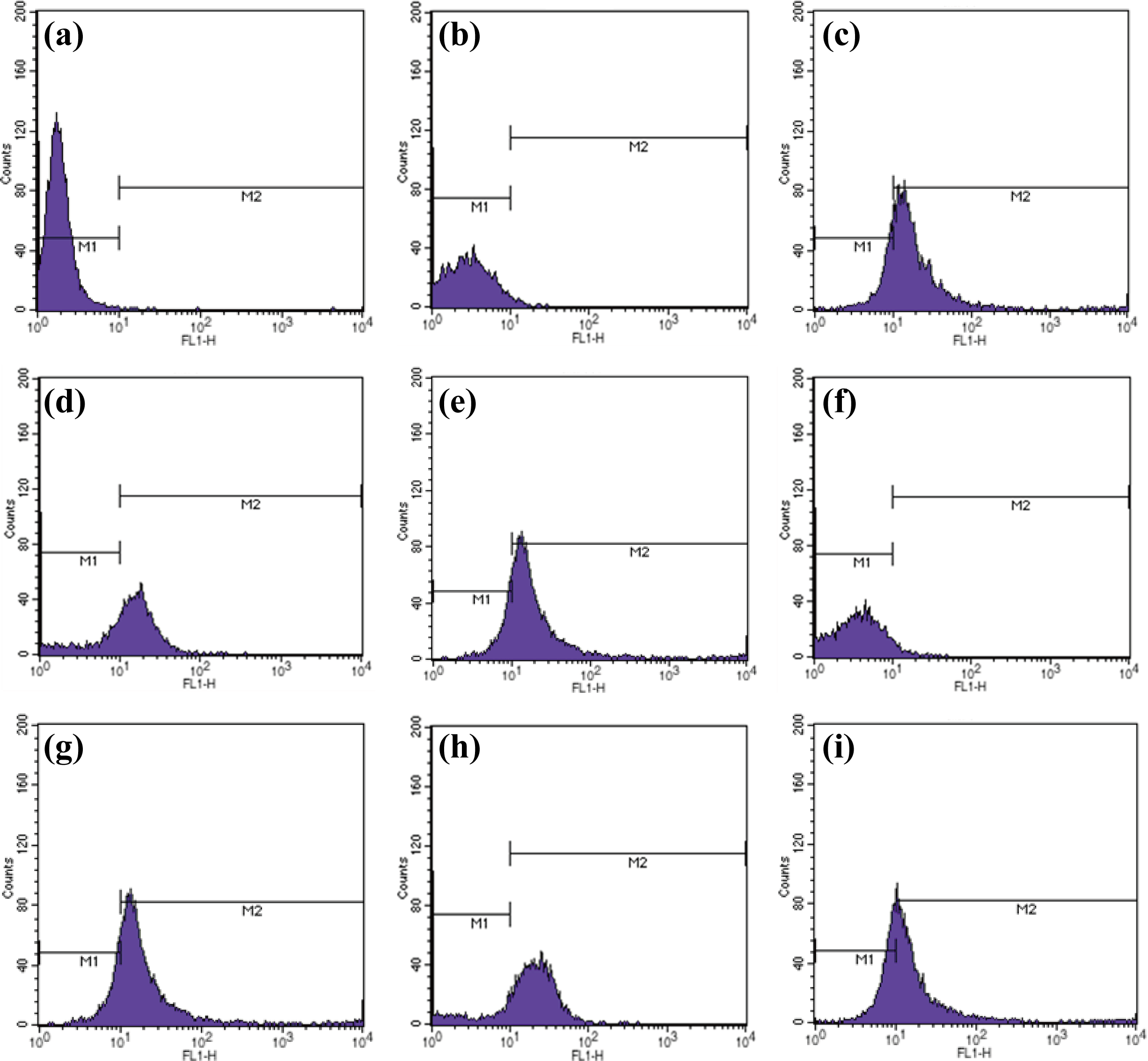


**Figure S4.** Flow cytometric results of HCCLM3 cells treated for 4 h with PBS buffer (a), {(Au^0^)_100_G5.NH_2_-FI-DOTA(Mn)-HA} NPs at Mn concentrations of 10 (b), 25 (c), 75 (d), or 100 (e) μg/mL, or CD44 receptor blocked at 10 (f), 25 (g), 75(h), or 100 (i) μg/mL.

**Figure S4**

**Wang et al.**
